# Transmission Electron Microscopy Study of Mitochondria in Aging Brain Synapses

**DOI:** 10.3390/antiox8060171

**Published:** 2019-06-11

**Authors:** Vladyslava Rybka, Yuichiro J. Suzuki, Alexander S. Gavrish, Vyacheslav A. Dibrova, Sergiy G. Gychka, Nataliia V. Shults

**Affiliations:** 1 Department of Pharmacology and Physiology, Georgetown University Medical Center, Washington, DC 20057, USA; Vor5@georgetown.edu (V.R.); ys82@georgetown.edu (Y.J.S.); 2 Department of Pathological Anatomy N2, Bogomolets National Medical University, Kiev 01601, Ukraine; alexander.gavrysh@gmail.com (A.S.G.); dibrova03@ukr.net (V.A.D.); gychka59@gmail.com (S.G.G.)

**Keywords:** aging, brain, electron microscopy, hippocampus, mitochondria, synapse

## Abstract

The brain is sensitive to aging-related morphological changes, where many neurodegenerative diseases manifest accompanied by a reduction in memory. The hippocampus is especially vulnerable to damage at an early stage of aging. The present transmission electron microscopy study examined the synapses and synaptic mitochondria of the CA1 region of the hippocampal layer in young-adult and old rats by means of a computer-assisted image analysis technique. Comparing young-adult (10 months of age) and old (22 months) male Fischer (CDF) rats, the total numerical density of synapses was significantly lower in aged rats than in the young adults. This age-related synaptic loss involved degenerative changes in the synaptic architectonic organization, including damage to mitochondria in both pre- and post-synaptic compartments. The number of asymmetric synapses with concave curvature decreased with age, while the number of asymmetric synapses with flat and convex curvatures increased. Old rats had a greater number of damaged mitochondria in their synapses, and most of this was type II and type III mitochondrial structural damage. These results demonstrate age-dependent changes in the morphology of synaptic mitochondria that may underlie declines in age-related synaptic function and may couple to age-dependent loss of synapses.

## 1. Introduction

Aging is a physiological, progressive, and time-dependent process that results in accumulated changes at the cellular and molecular levels. The brain is highly sensitive to the aging process, as many neurodegenerative diseases clearly show [1,2,3]. The hippocampus is especially vulnerable to damage at the early stage of aging [4], which results in the development of several age-dependent neurodegenerative disorders, including Parkinson’s and Alzheimer’s diseases [5,6].

The hippocampus is one of the best-studied structures in the human and animal brain [7,8,9,10]. While the age-related decline in hippocampal volume has been well documented, most knowledge of hippocampal relationships between structure and function has been discovered in the context of neurological and neurodegenerative diseases [11,12]. The relationship between cognitive aging and hippocampal structure in the absence of disease remains relatively understudied.

The human brain requires about 20% of the body’s total energy production to fulfill its function, which is the highest energy consumption of any organ. During aging, accumulated changes in brain cells impair energy metabolism, which leads to neurodegenerative disorders [13]. Mitochondria are primarily responsible for producing ATP via oxidative phosphorylation in the inner mitochondrial membrane. ATP is required for all energy-dependent cellular processes, such as the regulation of intracellular calcium homeostasis, synaptic plasticity, and the synthesis of neurotransmitters [14,15]. Aside from producing less ATP, damaged, and less functional mitochondria can induce the formation of reactive oxygen species with cellular toxicity [16,17].

Accumulated structural alterations to mitochondria with age contribute to the regulation of reduction–oxygenation (redox) homeostasis and lead to a decline in mitochondrial function [18]. This decline might contribute to the age-dependent decline in brain function [19,20,21]. To our knowledge, however, no detailed morphological studies have been performed on the structural changes to mitochondria in the aging hippocampus.

Using transmission electron microscopy, the present study reports detailed ultrastructural examinations of hippocampal synapses in young and old rats. Understanding the characteristics of synaptic changes during aging should lead to the development of therapeutic strategies for neurodegenerative disorders.

## 2. Materials and Methods

### 2.1. Experimental Animals

Male Fischer rats were purchased from Charles River Laboratories International. Groups of nine young-adult rats (10 months old) and nine older rats (22 months old) were compared. The Georgetown University Animal Care and Use Committee approved all animal experiments (ethical protocol code: 2017-0056), and the investigation conformed to the National Institutes of Health (NIH) Guide for the Care and Use of Laboratory Animals.

### 2.2. Transmission Electron Microscopy (TEM)

Brains were removed from the Fischer rats at 10 (*n* = 9) and 22 (*n* = 9) months of age. The ventral part of the hippocampus layer–the CA1 region was isolated and cut into ~1 mm^3^ cubes. Tissues were fixed in a solution of 4% paraformaldehyde and 0.5% glutaraldehyde/0.2 M cacodylate and then post-fixed with 1% osmium tetroxide and embedded in EmBed812. Ultrathin sections were post-stained with uranyl acetate and lead citrate and examined in a Talos F200X FEG transmission electron microscope at 80 kV located at the George Washington University Nanofabrication and Imaging Center. Low-magnification imaging was followed by high-magnification imaging. Representative images were acquired and recorded with TIA software.

### 2.3. Morphometric Analysis

Morphometric analyses of TEM images were performed with the Fiji Software on a sample of 15 systematically, uniformly, and randomly selected images. The total volume of the CA1 region of the hippocampal layer was estimated using point counting according to Cavalieri’s principle. The total number of synapses (310 from each group) was determined as the product of the total volume of the CA1 region of the hippocampus layer and the numerical density of synapses [22]. The total number of synaptic mitochondria (450 from each group) was determined by the morphometric technique using a dot grid [23]. The coefficient of energy efficiency of mitochondria (CEEM) was defined as the product of the number of mitochondrial cristae and the area of mitochondria [24]. The criteria for synaptic curvatures were defined as previously described [25]. The determination of the types of mitochondria has been described in Shults et al. [26].

### 2.4. Statistical Analysis

Means and standard errors were calculated. Comparisons between two groups were analyzed by a two-tailed Student’s t-test, and comparisons among three or more groups were analyzed by one-way analysis of variance (ANOVA) with a Student–Newman–Keuls post-hoc test using GraphPad Prism (GraphPad Software, Inc., La Jolla, CA, USA). *p* < 0.05 was considered significant.

## 3. Results

### 3.1. Synapses in the Hippocampus at 10 Months of Age

**Figure 1:**
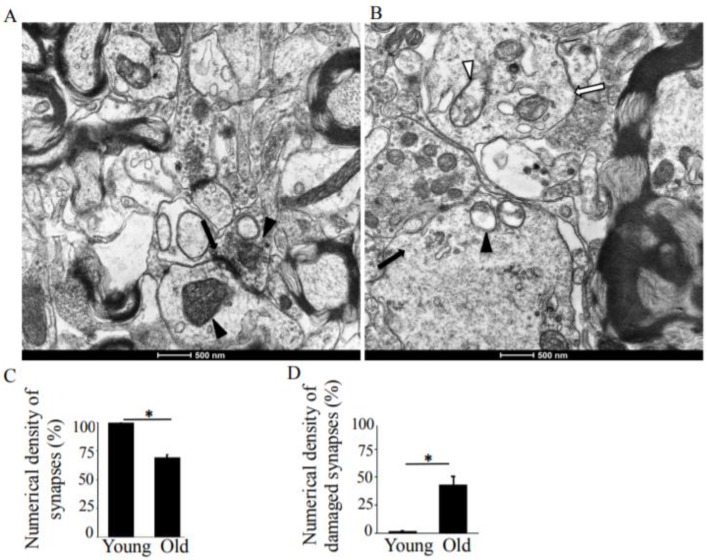
Transmission electron microscopy (TEM) analysis of the hippocampus, CA1 region in young-adult and old rats. (**A**) A representative TEM image of a young rat CA1 region, demonstrating normal synaptic structure. The pre- and post-synaptic densities are sharply defined and contain electron–dense materials that are uniformly distributed. The arrows show concave curvature of symmetric synapses. The arrowheads point to synaptic mitochondria with normal structure. Magnification ×14,000. (**B**) TEM image showing degenerative changes in the pre- and post-synaptic compartments. The arrows point to synapses. Magnification ×14,000. (**C**) Means ± SEM, percent numerical density of synapses in young-adult and old rats. (**D**) Means ± SEM, percent numerical density of damaged synapses in young-adult and old rats. * indicates significant differences at *p* < 0.05.

The ultrastructure of all synapses in the CA1 region of the hippocampus in young-adult rats were generally identical. The numerical density of synapses was 9.45 µm^2^, comprising 7.76 μm^2^ and 1.69 μm^2^ asymmetric and symmetric synapses, respectively (Table 1). In the pre-synaptic compartment, the vesicles are concentrated in the vicinity of the pre-synaptic membrane, forming the active zone. The synaptic vesicles are oval or round in shape and uniform in size. The post-synaptic densities are sharply defined, with uniformly distributed, electron-dense materials (Figure 1A). Most asymmetric synapses had concave curvature (Figure 1A, arrow), with significantly fewer flat and convex curvatures, as shown in Table 1. Synaptic mitochondria were usually oval in shape and varied from 0.3–0.8 µm in size, containing transversally oriented cristae (Figure 1A, arrowheads). In most cases, the matrices of pre-synaptic mitochondria were more electron-dense than those of post-synaptic mitochondria (Figure 1A). Thus, the ultrastructural organization of the mitochondria corresponded to type I.

### 3.2. Synapses in the Hippocampus at 22 Months of Age

In old rats, the synapses of the CA1 region of the hippocampus are characterized by significant degenerative changes in the pre- and post-synaptic compartments. The numerical density of synapses reduced from 9.45 µm^2^ to 6.67 µm^2^ (Table 1). The vesicles of the presynaptic sites were not concentrated in the vicinity of the pre-synaptic membrane. Degenerative changes in the presynaptic compartment from the “light type” of destruction, however, were also accompanied by the “dark type” as well (Figure 1B). The “light type” of destruction is characterized by edema and reduced electron density in the presynaptic compartments (Figure 1B, white arrow). The vesicles were distributed unevenly, without the formation of the active zone. In this case, destroyed mitochondria (Figure 1B, white arrowhead) accompanied a significant decrease in the number of synaptic vesicles. The “dark type” of destruction includes increasing osmiophilic of the pre- and post-synaptic compartments, swelling of mitochondria, and disruption to and glued osmiophilic vesicles (Figure 1B, black arrow, arrowhead).

In old rats, the synaptic vesicles connected with the synaptic membrane were reduced in number and disorganized and had blurred pre-synaptic membranes (Figure 1B). The shapes and sizes of the synaptic vesicles differed from the oval or round shapes in the young rats. In terms of their observed disorganization, the vesicles did not fill the pre-synaptic compartments. These characteristics of vesicle distribution observed in the rats are similar to Adams’ report in the human brain [27]. The total number of synapses decreased by 29.4% with increased age (Figure 1C), and the numerical density of the damaged synapses in old rats increased by 41.7% (Figure 1D).

**Figure 2:**
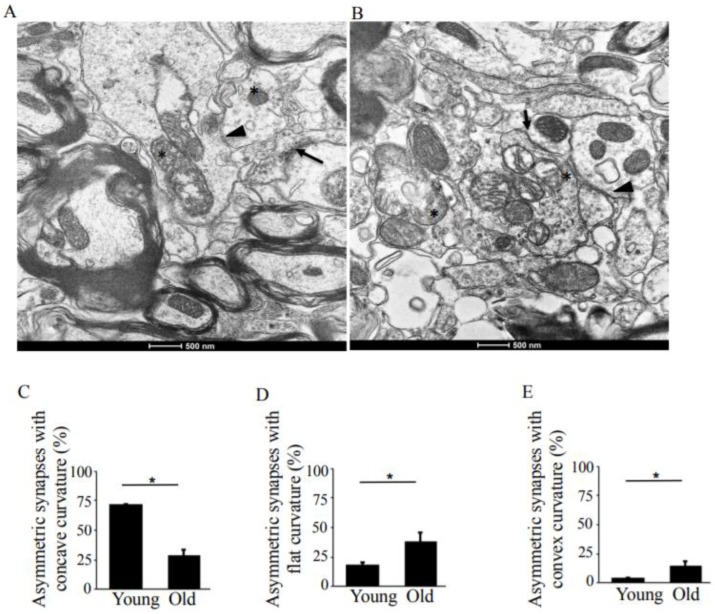
TEM analysis of asymmetric synapses in the hippocampus, CA1 region in young-adult and old rats. (**A**) TEM image of young-adult rat, CA1 region. The arrows point to the asymmetric synapses with concave curvature, whereas the arrowheads indicate asymmetric synapses with convex curvature. The asterisks indicate synaptic mitochondria containing normal cristae. Magnification ×14,000. (**B**) TEM image of old rat, CA1 region. The arrows points to asymmetric synapses with concave curvature, whereas the arrowheads indicate asymmetric synapses with flat curvature. The asterisks indicate damaged synaptic mitochondria. Magnification ×14,000. (**C**) Means ± SEM, percent asymmetric synapses with concave curvature in young-adult and old rats. (**D**) Means ± SEM, percent asymmetric synapses with flat curvature in young-adult and old rats. (**E**) Means ± SEM, percent asymmetric synapses with convex curvature in young-adult and old rats. * indicates significant differences at *p* < 0.05.

While the total number of asymmetric synapses (Figure 2A,B, arrow) in old rats was comparable to the number in young rats, the number of asymmetric synapses with concave curvature decreased to 1.89 µm^2^ from 5.6 µm^2^ (Table 1), a reduction to 28.3% from 72.1% with age (Figure 2C). The number of asymmetric synapses with flat and convex curvatures (Figure 2A,B, arrowheads) increased by 19% and 10%, respectively (Figure 2D,E).

**Figure 3:**
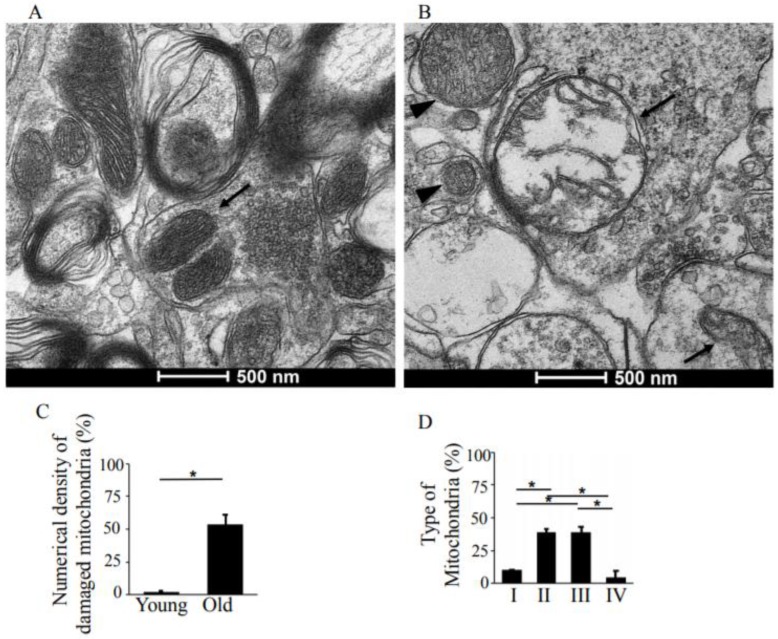
TEM analysis of structural changes in synaptic mitochondria of the hippocampus, CA1 region, in young-adult and old rats. (**A**) Representative TEM image of young-adult rats, CA1 region, demonstrating normal structure of the synaptic mitochondria (arrow). Magnification ×33,000. (**B**) TEM image of old rat showing damaged synaptic mitochondria. Arrowhead points to type II mitochondrial damage, while the arrows indicate type III mitochondrial damage. Magnification ×33,000. (**C**) Means ± SEM, percent numerical density of damaged mitochondria in young-adult and old rats. (**D**) Means ± SEM, percent type of mitochondria in the synapses of old rats. * indicates significant differences at *p* < 0.05.

**Figure 4:**
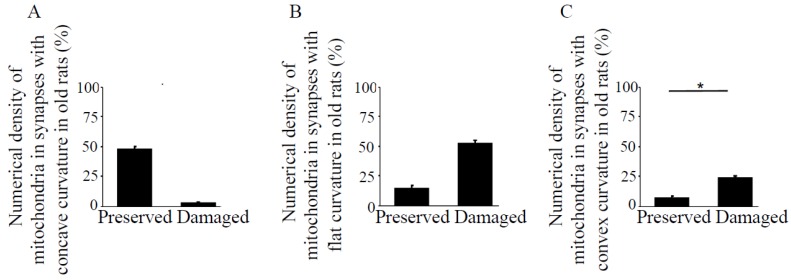
Numerical density of preserved and damaged mitochondria in synapses with different types of curvature in the hippocampus, CA1 region in old rats. Bar graphs represent means ± SEM of percent numerical density of mitochondria in the synapses with (**A**) concave, (**B**) flat, and (**C**) convex curvatures in old rats. * indicates significant differences at *p* < 0.05.

**Figure 5:**
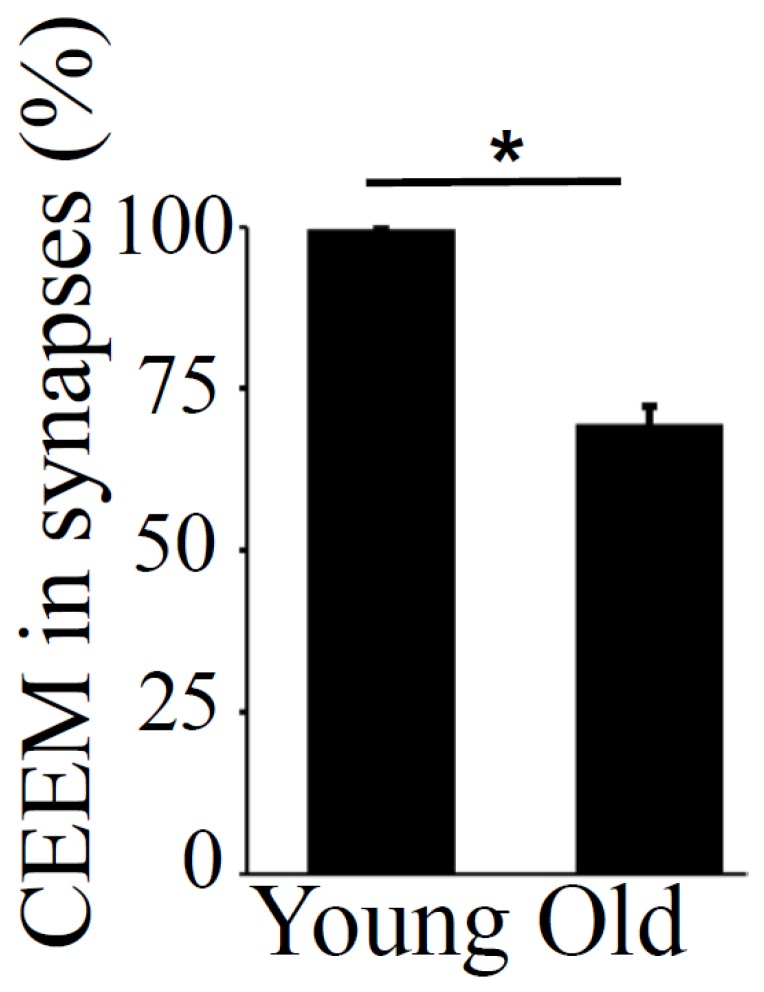
Coefficient of energy efficiency of synaptic mitochondria (CEEM) in the hippocampus, CA1 region, in young-adult and old rats. Means ± SEM, percent CEEM in synapses in young-adult and old rats. * indicates significant differences at *p* < 0.05.

The pre- and post-synaptic mitochondria in the damaged synapses of aging rats were characterized as abnormal, with non-distinct shape, non-uniform size, or both (Figure 2A,B, asterisks). Pre-synaptic mitochondria were less often increased in size than were mitochondria in the post-synaptic compartment. In both synaptic compartments, swollen, homogenized, and whirled cristae were observed, corresponding to type II mitochondrial damage (Figure 3B, arrowheads), whereas the mitochondrial structure in the synapses of young rats was normal or type I (Figure 3A, arrow). Compared to presynaptic mitochondria, post-synaptic mitochondria exhibited more severe degenerative changes. Crista homogenization and fragmentation in a significantly swollen electron–lucent matrix were observed in most post-synaptic compartments that corresponded to type III mitochondrial damage (Figure 3B, arrows). Type IV damaged mitochondria, with disrupted and discontinuous outer membranes and deficient in cristae, were minimal in both synaptic compartments. The total numerical density of damaged mitochondria in the synapses of old rats was up to 51.6% higher compared to young rats (Figure 3C). Most mitochondrial structural damage in the old rats were of the types II and III (Figure 3D). Morphometric analysis revealed that asymmetric synapses with concave curvature contained 48% of the mitochondria with preserved structure (Figure 4A), while synapses with flat curvature contained 53% of the damaged mitochondria (Figure 4B), and synapses with convex curvature contained 24% of the damaged mitochondria (Figure 4C). Morphometric analysis of the TEM images further revealed that energy production by the synaptic mitochondria, as determined by calculating the CEEM of the CA1 region of the hippocampus, reduced by 42.4% (Figure 5) in old compared to young-adult rats.

## 4. Discussion

The present study was designed to investigate age-related ultrastructural changes in the synapses and synaptic mitochondria of the CA1 region of the hippocampal layer in young-adult and old rats by transmission electron microscopy. We identified degenerative changes in mitochondria that are relevant to age-dependent synaptic damage. As has been well-documented, aging is accompanied by a decline in mitochondrial function, which might contribute to the age-dependent decline in brain function [28,29]. However, no age-dependent ultrastructural changes in hippocampal synapses have been defined.

The earlier study showed elevated markers of oxidative stress in the hippocampus that correlated with a reduction in the number of synapses and the disruption of neurotransmitter transport [30]. The mitochondrial dysfunction caused by Ca^2+^ that seems to initiate mitochondrial failure also contributes to the synaptic deficit observed during aging [31,32]. The mitochondria are organelles that produce ATP, and their function is closely related to the mitochondrial ultrastructure. The outer and inner mitochondrial membranes enclose and define the inter-membrane space and matrix compartments. Invagination of the inner membrane forms cristae, where the respiratory chain complex is situated, which in synapses, directly engages in mitochondrial function. Disturbance to the mitochondrial crista ultrastructure thus contributes to deficits in ATP synthesis [33].

Mitochondrial reconstruction in hippocampal synapses during aging alters synaptic function, which is determined by the number of symmetric and asymmetric synapses. An earlier study determined that synapses with flat curvature are inactive, whereas synapses with curvature are in an active, functional state, and a concave curvature indicates exocytosis [34]. The asymmetric synapses with concave curvature contain mitochondria with preserved outer and inner membranes. Notably, mitochondria in the pre-synaptic compartments are smaller and higher in the intensity of electron-dense material, which exhibits more destructive changes with age, than post-synaptic mitochondria.

With age, the CA1 region of the hippocampus not only loses a number of synapses but also experiences damaged ultrastructural synaptic architecture accompanied by damaged mitochondria. The present study demonstrated that the number of asymmetric synapses with concave curvature containing preserved mitochondria decreases during aging, while asymmetric synapses with flat curvature, that contain damaged mitochondria become dominant. Homogenization and fragmentation of mitochondrial cristae in a swollen electron–lucent matrix, which results in sustained energy demand, may decrease synaptic function of the CA1 region in the hippocampus and reduce plasticity of the synaptic contact zone, which manifests as deficits in memory.

## 5. Conclusions

The present transmission electron microscopy study demonstrates that a decline in the ultrastructural quality of mitochondria in the synapses of the CA1 region of the hippocampus is associated with age-related damage to synapses and their reduction in numerical density. This new understanding of the pathological mechanism of age-dependent destruction of synaptic mitochondria in association with synaptic damage and loss in the CA1 region of the hippocampus should be useful for designing therapeutic strategies to prevent or treat neurodegenerative disorders related to aging.

## Figures and Tables

**Table 1:** Numerical density of synapses in the hippocampus, CA1 region (×10^8^/mm^3^).

Fisher Rats	Total	Symmetric	Asymmetric			
			Total	Flat Curvature	Concave Curvature	Convex Curvature
10 months	9.45 ± 0.9	1.69 ± 0.2	7.76 ± 0.2	1.78 ± 0.01	5.6 ± 0.01	0.38 ± 0.05
22 months	6.67 ± 1.5	1.28 ± 1.9	5.39 ± 0.5	2.53 ± 0.07	1.89 ± 0.02	0.97 ± 0.09
